# Machine Learning on Low-Cost Edge Devices for Real-Time Water Quality Prediction in Tilapia Aquaculture

**DOI:** 10.3390/s25196159

**Published:** 2025-10-04

**Authors:** Pinit Nuangpirom, Siwasit Pitjamit, Veerachai Jaikampan, Chanotnon Peerakam, Wasawat Nakkiew, Parida Jewpanya

**Affiliations:** 1 Department of Industrial Education and Technology, Faculty of Engineering, Rajamangala University of Technology Lanna, Chiang Mai 50300, Thailand; elecpnt@rmutl.ac.th; 2 Department of Industrial Engineering, Faculty of Engineering, Rajamangala University of Technology Lanna, Chiang Mai 50300, Thailand; siwasit.pitjamit@rmutl.ac.th; 3 Department of Technology Preparatory Education, College of Integrated Science and Technology, Rajamangala University of Technology Lanna, Chiang Mai 50300, Thailand; veerachai@rmutl.ac.th; 4 Department of Robotics Engineering and Artificial Intelligence Program, Faculty of Engineering, Chiang Mai University, Chiang Mai 50200, Thailand; chanotnon.pee@cmu.ac.th; 5 Department of Industrial Engineering, Faculty of Engineering, Chiang Mai University, Chiang Mai 50200, Thailand; wasawat@eng.cmu.ac.th

**Keywords:** Edge AI, ESP32, water quality, tilapia, MLR model, industry, innovation and infrastructure

## Abstract

This study presents the deployment of Machine Learning (ML) models on low-cost edge devices (ESP32) for real-time water quality prediction in tilapia aquaculture. A compact monitoring and control system was developed with low-cost sensors to capture key environmental parameters under field conditions in Northern Thailand. Three ML models—Multiple Linear Regression (MLR), Decision Tree Regression (DTR), and Random Forest Regression (RFR)—were evaluated. RFR achieved the highest accuracy (R^2^ > 0.80), while MLR, with moderate performance (R^2^ ≈ 0.65–0.72), was identified as the most practical choice for ESP32 deployment due to its computational efficiency and offline operability. The system integrates sensing, prediction, and actuation, enabling autonomous regulation of dissolved oxygen and pH without constant cloud connectivity. Field validation demonstrated the system’s ability to maintain DO within biologically safe ranges and stabilize pH within an hour, supporting fish health and reducing production risks. These findings underline the potential of Edge AIoT as a scalable solution for small-scale aquaculture in resource-limited contexts. Future work will expand seasonal data coverage, explore federated learning approaches, and include economic assessments to ensure long-term robustness and sustainability.

## 1. Introduction

Tilapia farming is a significant economic activity for Thai farmers, particularly in the northern and central regions, where natural water resources support aquaculture. Tilapia is valued for its tolerance to diverse environments, rapid growth, and strong market demand. However, water quality remains a major constraint that affects survival and growth. Seasonal and climatic fluctuations can reduce dissolved oxygen (DO), particularly during rainy or cloudy periods; sometimes, DO falls below critical thresholds and causes stress, hypoxia, or even fish mortality through surfacing behavior (“air gulping”) [1]. Sudden shifts in pH and temperature from heavy rainfall further disrupt homeostasis, impairing ion regulation and ammonia excretion and increasing vulnerability to disease [2].

Studies have shown that DO concentrations below 5 mg/L impair tilapia growth and increase mortality risk, particularly in high-density systems without proper aeration [2]. Climate variability, including heavy rainfall and drought, can further destabilize pond ecosystems, leading to ammonia or organic pollutant buildup that is toxic to fish [3]. These conditions threaten long-term growth and reproduction, underscoring the need for effective water quality management through continuous monitoring of DO, pH, temperature, and ammonia, together with adaptive planning based on seasonal and meteorological conditions.

In recent years, the Internet of Things (IoT) has become a transformative tool in aquaculture, enabling real-time monitoring of water quality, a key factor for fish health and growth. Flores-Iwasaki et al. reviewed IoT-based systems in Biofloc, RASs, and aquaponics, showing that pH, temperature, and DO sensors were most common, effectively reducing mortality and improving growth [4]. Olanubi et al. demonstrated a smart water quality control system using ESP32 with pH, temperature, and turbidity sensors, transmitting data to the cloud and providing real-time alerts via mobile apps [5]. While these advances support responsive and semi-autonomous control, Bonfante Rodríguez et al. noted persistent challenges in rural or small-scale farms, including unstable connectivity, hardware unsuited for harsh conditions, and limited sensor precision—barriers that hinder full adoption of autonomous IoT systems [6].

To address the limitations of IoT systems focused mainly on sensing and visualization, recent studies have integrated Artificial Intelligence (AI) and Machine Learning (ML) into aquaculture. These technologies improve predictive capabilities for key parameters such as DO, pH, temperature, and ammonia, directly affecting fish health and survival. Baena-Navarro et al. developed a hybrid system combining ML models, IoT sensors, and the Quantum Approximate Optimization Algorithm (QAOA), which cut training time by up to 50% while maintaining survival rates above 90% under volatile tropical conditions [7]. A major advantage of this approach is its dual capability for both local and cloud-based processing, making it suitable for large-scale as well as resource-limited farms.

Roy and Kumari reviewed the application of AI in Recirculating Aquaculture Systems (RASs) and aquaponics, emphasizing predictive analytics for optimizing DO, pH, and nutrient levels. These AI-driven systems enhance resource efficiency and promote sustainable production [8]. Most, however, remain cloud-dependent, requiring high deployment costs and stable internet connectivity, which are often inaccessible to smallholder farmers. To overcome these limitations, Esty et al. introduced EcoGuard, an IoT platform leveraging edge computing and federated learning for distributed processing. This system enables timely water quality predictions and mobile alerts while ensuring data privacy and reducing costs, making it more accessible and scalable for small- and medium-scale farms [9].

From the literature reviewed [1,2,3,4,5,6,7,8], it appears that most Smart Aquaculture systems remain largely passive, collecting sensor data and transmitting it to the cloud with minimal or no deployment of predictive ML models on microcontrollers. Existing studies rarely consider endpoint constraints—such as limited processing power, memory, energy, and intermittent connectivity—that restrict timely responses and hinder effective real-time water quality control. Furthermore, many reported models and thresholds are derived from nonlocal contexts, making them difficult to adapt for site-specific decision-making and insufficient to capture heterogeneity in geomorphology, hydrochemistry, and climate across ponds.

Accordingly, the key research gap lies in the absence of practical demonstrations that deploy predictive ML models directly on ultra-low-cost edge devices, such as the ESP32, for aquaculture water quality management. To address this gap, the present study develops and validates an Edge AIoT system for tilapia aquaculture in Northern Thailand (Doi Lo District, Chiang Mai Province). The system integrates IoT sensing with ML deployed locally on the ESP32 to predict dissolved oxygen (DO) and pH without continuous cloud dependency. As one of the first Edge AI applications in aquaculture, we introduce an explicit “accuracy–feasibility” trade-off framework for model selection under edge constraints. Also, we provide field evidence that lightweight Multiple Linear Regression (MLR) running on ESP32 can maintain DO and pH within biologically optimal ranges at a low energy cost. The edge models are further coupled with actuators (e.g., aeration pumps, localized heaters) and a user alerting mechanism for pH adjustment, enabling autonomous, real-time water quality regulation, reducing production risks, and advancing the sustainability of tilapia aquaculture. This study’s novelty lies in it being among the first studies to demonstrate predictive ML deployment on ultra-low-cost microcontrollers for aquaculture, as well as advancing the shift from cloud-dependent monitoring to useful, independent, and scalable Edge AIoT systems by presenting a realistic accuracy–feasibility architecture and verifying its performance in the actual world.

## 2. Literature Review

Recent studies have increasingly emphasized the convergence of Internet of Things (IoT) and Machine Learning (ML) for water quality monitoring. Advances in Machine Learning and IoT for Water Quality Monitoring [10] provided a broad overview, highlighting how IoT enables continuous environmental sensing while ML enhances pattern recognition and forecasting accuracy of critical parameters. The review also examined wireless communication technologies such as LPWAN, Wi-Fi, and ZigBee, concluding that IoT–ML integration represents a promising direction for intelligent monitoring. In practice, An IoT Real-Time Potable Water Quality Monitoring Model [11] implemented Arduino and NB-IoT for real-time water quality monitoring, achieving high classification accuracy and user alerts; however, it lacked automated feedback or control.

Efforts to improve system responsiveness have further explored distributed computation. Intelligent Edge–Cloud Framework for Water Quality Monitoring [12] demonstrated that edge processing substantially reduces latency and energy consumption compared to cloud-only approaches, while hybrid architectures enhance accuracy. These findings confirm the growing interest in computational designs that move analytics closer to the data source.

Parallel advances have also been made in predictive modeling of dissolved oxygen (DO). The Development of Dissolved Oxygen Forecast Model Using Hybrid ML [13] integrated hydro-meteorological variables with advanced algorithms, achieving high precision, while Using Machine Learning Models for Short-Term Prediction of DO in a Microtidal Estuary [14] demonstrated that temporal sequence models such as RNN and LSTM are highly effective for multi-week predictions. Despite their accuracy, these studies were based on meteorological or laboratory data rather than IoT-enabled real-time deployment, limiting direct application to aquaculture ponds.

Within aquaculture contexts, IoT-enabled platforms have begun to emerge. *AquaBot* [15] employed ESP32-based sensing combined with ML classifiers to recommend fish species based on prevailing conditions. While innovative, this system focused on recommendations rather than direct control of water parameters. Likewise, Random Forest-Based Framework for Water Quality Prediction [16] applied ML to inland and coastal waters using in situ and satellite data, but it operated entirely on server-based computation and lacked real-time aquaculture applicability. A systematic review of 217 studies [17] further confirmed that IoT applications in aquaculture are dominated by monitoring pH, temperature, and DO but face persistent challenges in automated control, sensor maintenance, and local ML deployment.

As summarized in Table 1, these representative studies highlight three clear patterns:

- IoT systems are often limited to data collection with minimal local intelligence [11,12,15].
- ML prediction models achieve high accuracy [13,14] yet remain disconnected from real-time aquaculture operations.
- Although edge computing frameworks reduce latency and energy use [12], small-scale aquaculture ponds have not yet benefited from their deployment.

Taken together, the literature [10,11,12,13,14,15,16,17] demonstrates substantial progress in IoT–ML integration for water quality management. However, most implementations remain cloud-dependent, incur high costs, or fail to account for endpoint resource constraints such as limited processing power, energy, and intermittent connectivity. Importantly, none of the reviewed works show predictive ML deployment directly on ultra-low-cost microcontrollers such as ESP32 for aquaculture. This persistent gap motivates the present study, which develops and validates an Edge AIoT system for real-time prediction and autonomous control of dissolved oxygen and pH under field conditions. To address this, the following section details the system design, data acquisition, and model evaluation protocols.

## 3. Materials and Methods

To develop a real-time, automated water quality control system for tilapia farming using a low-cost platform, this study designed and deployed a prototype system in a real-world aquaculture setting. The system integrates environmental and water quality sensors with machine learning-based prediction algorithms and automatic control mechanisms. Emphasis was placed on ensuring that the system can operate effectively under limited infrastructure and intermittent internet connectivity. The details of the implementation are as follows:

### 3.1. Experimental Area and Data Collection

**Figure 1:**
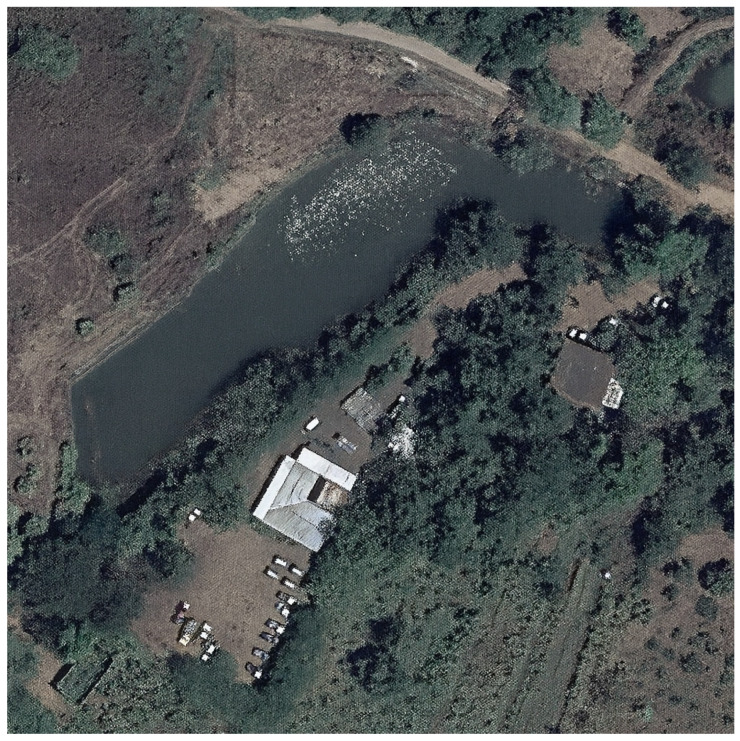
Experimental area.

This study was conducted in a real-world field setting at the Agricultural Learning and Productivity Enhancement Center in Doi Lo District, Chiang Mai Province, Thailand. The site is located within a mixed-agriculture zone characterized by favorable climatic and environmental conditions for integrated aquaculture. The geographic coordinates of the study location are 18.4947° N, 98.7771° E, as shown in Figure 1.

The experimental site consists of a 1000-square-meter open-air tilapia pond equipped with a custom-designed floating platform. This platform hosts solar panels for a renewable power supply and supports the deployment of various water and environmental sensors. An ESP32-based microcontroller serves as the central processing unit, offering integrated Wi-Fi and Bluetooth connectivity.

**Figure 2:**
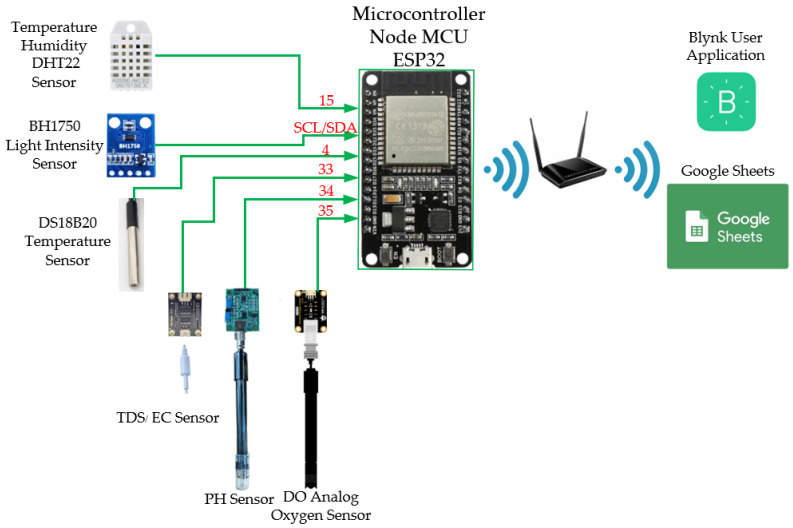
Architecture of water quality measurement system.

A suite of sensors was installed on platform [18], including air and water temperature sensors, a relative humidity sensor, a light intensity sensor (BH1750), and probes for electrical conductivity/total dissolved solids (EC/TDS), as well as dedicated sensors for dissolved oxygen (DO) and pH levels. The ESP32 microcontroller—powered by a dual-core Xtensa LX6 processor running at up to 240 MHz with 4 MB of flash memory and 512 kB of RAM—enables on-device data processing and supports lightweight machine learning inference without reliance on external servers, as shown in Figure 2.

**Figure 3:**
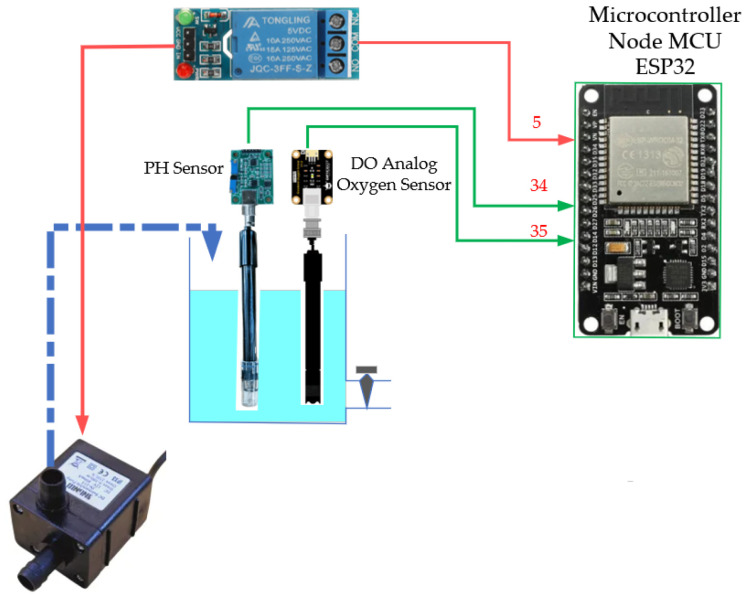
pH measurement system and dissolved oxygen sensor.

The DO and pH sensing system was designed to sample every hour, balancing data resolution and sensor longevity. Water is pumped into a 900 mL chamber via a submersible pump and relay control to minimize air bubble interference with the electrochemical DO probe. A 5 min stabilization delay ensures accurate readings, followed by drainage within 45 min to prevent sediment buildup. A 1 cm water level is retained to maintain sensor hydration, as recommended by the manufacturer [18]. To further ensure data reliability, both sensors were factory-calibrated before deployment and recalibrated in the field at two-week intervals. The pH sensor was adjusted using three standard buffer solutions (pH 4.0, 7.0, and 10.0), while the DO sensor employed a two-point method with air-saturated water and sodium sulfite solution for zero-oxygen reference, as shown in Figure 3.

To reduce system complexity and improve data accuracy, the water quality monitoring system is designed to operate in hourly cycles. An ESP32 board controls the water intake into the sampling chamber and then waits for probe stabilization before measuring dissolved oxygen (DO), pH, and electrical conductivity/total dissolved solids (EC/TDS). Other parameters, including air temperature, relative humidity, light intensity, and water temperature, are directly measured using environmental sensors connected to the ESP32. All collected data are transmitted to a dashboard and cloud server for real-time visualization. The core system logic is summarized in the following pseudocode:

every 1 h:
  pump water into chamber (12 s), wait to stabilize (5 min)
  read sensors: air temp/RH, light, water temp, EC
  read ADC: pH → voltage → pH value
  read ADC: DO → mV → ppb → mg/L with correction
  read rolling ADC buffer: TDS → voltage → nonlinear calc
  send all values to Blynk and Google Sheets
continuously:
  sample TDS every 40 ms → store in circular buffer

**Figure 4:**
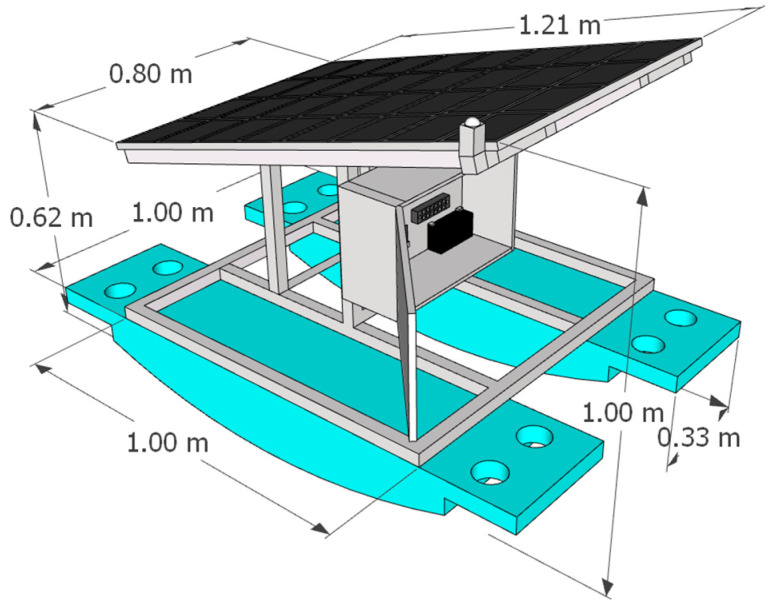
Floating platform structure for water quality monitoring.

The water quality monitoring system is built on a 1 × 1 m floating platform, featuring a control box, solar panel, and sensor chamber placed in a still-water channel to minimize wave interference. The structure is made of galvanized steel or aluminum and mounted on two pontoons, anchored by rope and concrete blocks. A 12 V solar panel and battery supply power via an IP65 control box. The system includes grounding to prevent signal noise, especially for pH and DO probes, ensuring long-term operation in tilapia aquaculture settings. The floating platform structure is shown in Figure 4.

### 3.2. Machine Learning Model Development

**Figure 5:**
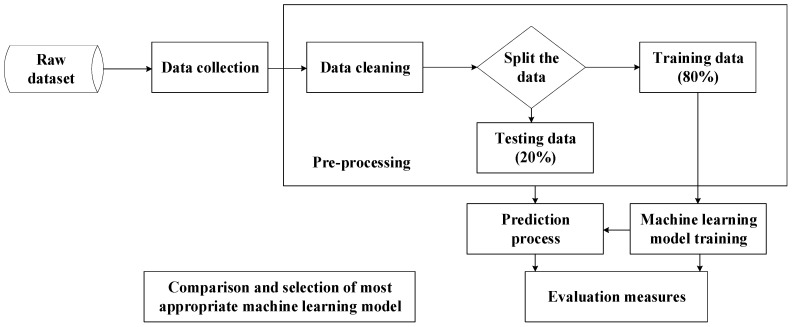
ML model development workflow.

The development process of the environmental control algorithm for tilapia farming using machine learning is illustrated in Figure 5. It began with collecting raw data from sensors and other sources, followed by organizing and cleaning the dataset. During preprocessing, outliers were detected using the interquartile range (IQR) method (values outside [Q1 − 1.5 IQR, Q3 + 1.5 IQR]) and removed. Missing values representing less than 5% of the dataset were imputed using linear interpolation, while larger gaps were excluded. Sensor calibration procedures were applied prior to data integration, as described in Section 3.1, to ensure the reliability of input variables.

The processed dataset was then divided into training (80%) and testing (20%) subsets to develop ML models, including Multiple Linear Regression (MLR), Decision Tree Regression (DTR), and Random Forest Regression (RFR). For DTR and RFR, hyperparameter tuning was conducted using grid search with five-fold cross-validation to improve robustness and avoid overfitting. Key parameters such as maximum depth, minimum samples per split, and the number of estimators were optimized based on validation performance. Model performance was evaluated using RMSE, MAE, and R^2^ [19].

The most suitable model was subsequently deployed on an ESP32 microcontroller by converting its parameters into C/C++ code. The embedded system included on-device normalization and real-time prediction of DO and pH values to control actuators such as aeration pumps, acid dosing systems, and water heaters.

### 3.3. Automatic Control System

**Figure 6:**
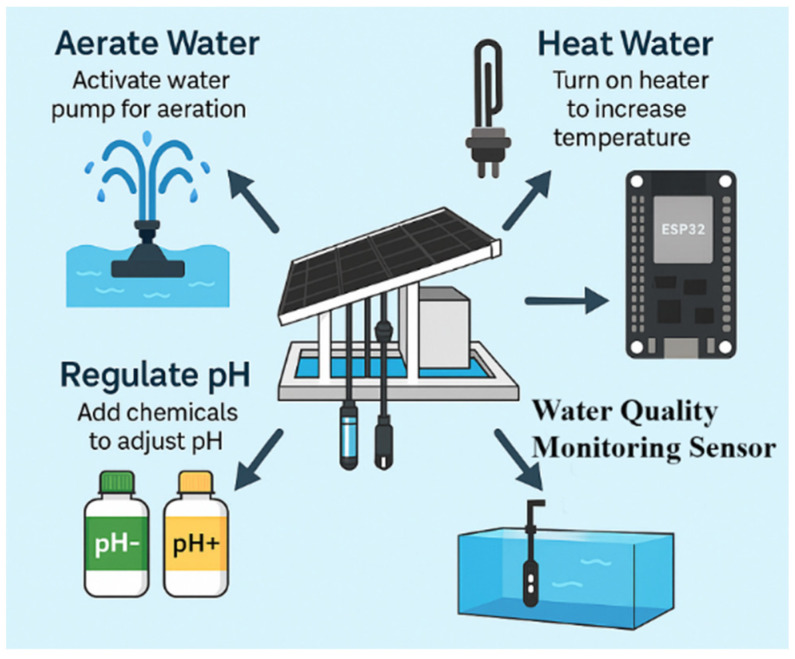
Smart water quality control system.

The water quality control system, as illustrated in Figure 6, was designed to respond proactively to environmental changes in the aquaculture pond. The control process begins with dissolved oxygen (DO) regulation: when DO drops below 6 mg/L, an adjustable aerator is automatically activated until levels rise to the optimal range of 6.2–6.4 mg/L. For pH control, if the measured value exceeds 8.0, a weak acid dosing pump operates in a stepwise manner to gradually bring the pH into the safe range of 7.0–8.0, with overshoot protection in place. Additionally, a 500-watt heater powered by a solar inverter is used to raise the water temperature locally near the floating platform by 1–2 °C, helping to stimulate schooling behavior in tilapia during cooler early morning hours [20].

## 4. Results

**Figure 7:**
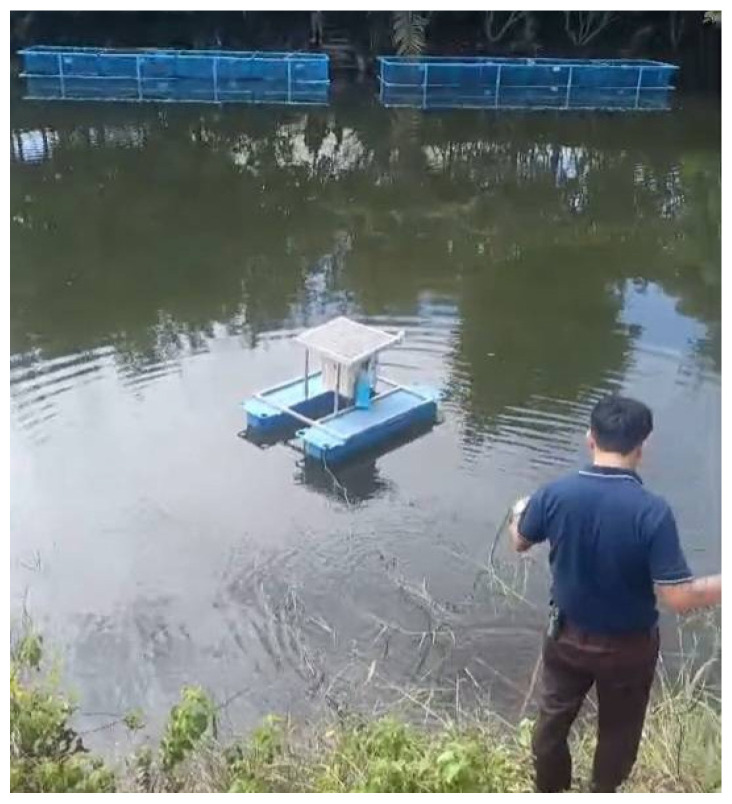
Real-world deployment of smart water system.

This system is designed and built to collect accurate and continuous water quality data. The collected data is used to develop and train machine learning models for predicting key parameters such as dissolved oxygen (DO), pH, and solute concentration. This approach enhances the efficiency of fish farming while reducing long-term reliance on costly sensors. The prototype and installation setup are shown in Figure 7.

### 4.1. Results of Water Quality Parameter Measurements

**Figure 8:**
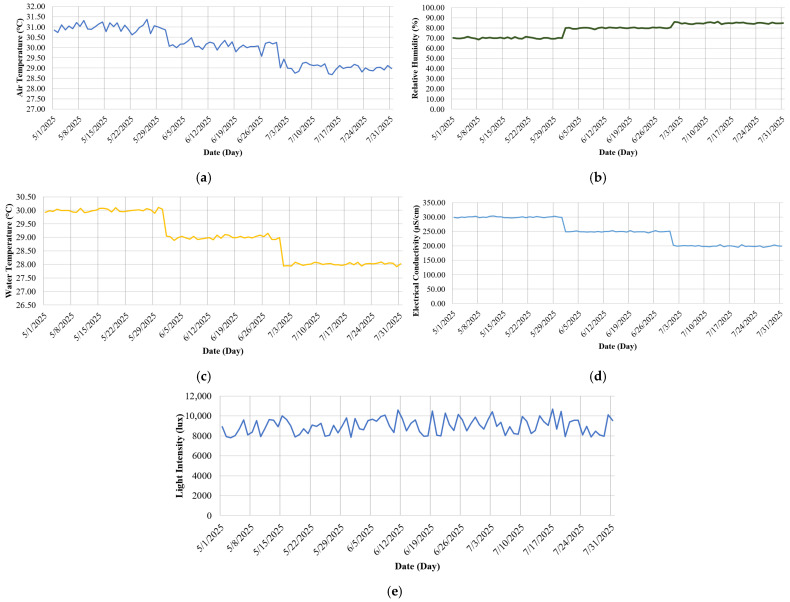
Environmental parameters recorded by the water quality monitoring system: (**a**) air temperature (°C), (**b**) relative humidity (%), (**c**) water temperature (°C), (**d**) electrical conductivity (μS/cm), and (**e**) light intensity (lux).

To assess the environmental conditions of the tilapia pond and monitor trends in parameters that may affect fish behavior and growth, the water quality monitoring system developed in this study was deployed in the target area. Continuous data collection was carried out from May to July 2025. The collected data were analyzed as daily averages for each environmental parameter, allowing for a clear visualization of trends over time. Five environmental parameters were monitored: air temperature, relative humidity, water temperature, electrical conductivity, and light intensity. The measurement results are presented in Figure 8.

Figure 8 illustrates the temporal variations in five key environmental parameters monitored from 1 May to 31 July 2025. As shown in Figure 8a, the average air temperature exhibited a gradual decrease from approximately 31.5 °C to 28.5 °C, corresponding with typical seasonal transitions. Figure 8b indicates a progressive increase in relative humidity from around 80% to nearly 95%, which is characteristic of the rainy season and may influence evaporation rates within the pond system. Figure 8c demonstrates a downward trend in water temperature, decreasing from approximately 29.5 °C to 27.5 °C, in alignment with the decline in ambient air temperature. This thermal change may impact fish behavior, particularly their tendency to aggregate in warmer microenvironments [21]. Notably, stepwise decreases observed during specific periods coincided with episodes of heavy rainfall, which rapidly introduced cooler water into the pond and contributed to abrupt thermal shifts. Figure 8d shows a reduction in electrical conductivity, dropping from about 320 µS/cm to 220 µS/cm, suggesting a dilution of dissolved salts and minerals, potentially due to increased rainfall or inflow [22]. Since the EC sensor used in this study included automatic temperature compensation (ATC), the stepwise decline is interpreted primarily as an effect of ion dilution caused by rainfall, rather than temperature alone. Lastly, Figure 8e presents the daily average light intensity, which remained relatively stable within the range of 8000–10,000 lux, indicating consistent light exposure throughout the observation period [23].

**Figure 9:**
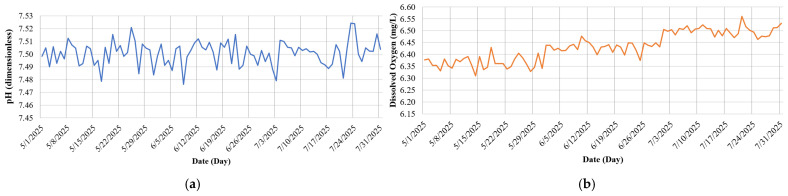
Water quality parameters monitored in the tilapia pond: (**a**) pH level, (**b**) dissolved oxygen (DO) concentration (mg/L).

Figure 9 presents the daily average measurements of key water quality parameters in the tilapia pond from May to July 2025. Figure 9a shows that the pH level remained relatively stable, fluctuating within a narrow range of 7.48 to 7.52, with an overall average of approximately 7.50—indicating favorable conditions for aquatic life [24]. Figure 9b illustrates a slight upward trend in dissolved oxygen (DO) concentration, increasing from around 6.30 to 6.55 mg/L, reflecting an environment conducive to the health and growth of tilapia [25].

### 4.2. Data Processing and Analysis

The data preparation process was systematically conducted to ensure suitability for analysis and forecasting. Key steps included schema validation, hourly resampling for timestamp normalization, and averaging duplicated entries within identical time intervals. Missing values were handled using context-aware techniques such as forward filling, linear interpolation, or segmentwise imputation depending on the variable dynamics. Outliers were detected using the interquartile range (IQR) method with parameter-specific thresholds based on water quality standards [26]. These preprocessing steps enhanced the reliability and readiness of the dataset for subsequent machine learning modeling. The results of the data preprocessing are presented in Table 2.

Table 2 presents the number and percentage of missing values across seven environmental variables—air temperature, humidity, light intensity, water temperature, electrical conductivity, pH, and dissolved oxygen—before and after preprocessing. Initially, the proportion of missing data ranged from 2.93% to 4.51%. Following imputation, all missing values were resolved, resulting in complete datasets ready for analysis.

Table 3 presents the summary of descriptive statistics for environmental and water quality parameters. ATemp represents air temperature (°C), RH is relative humidity (%), Light refers to light intensity (lux), WTemp is water temperature (°C), EC denotes electrical conductivity (µS/cm), pH indicates the water’s acidity/alkalinity level, and DO is dissolved oxygen (mg/L). All variables are summarized using daily hourly data (n = 2208). The data exhibits reasonable variability with no extreme outliers. Notably, DO ranged between 5.28 and 7.59 mg/L, while pH values were maintained within the optimal range of 6.87–8.14. Light intensity showed high fluctuation due to diurnal changes, and EC values were relatively stable around 250 µS/cm.

**Figure 10:**
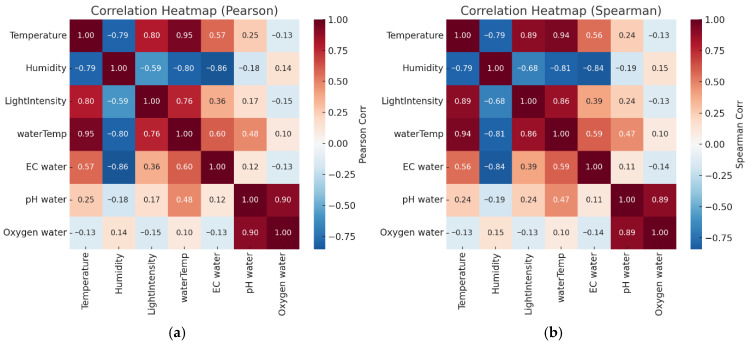
Correlation matrices of environmental and water quality variables using (**a**) Pearson correlation and (**b**) Spearman rank correlation.

Figure 10 illustrates correlation coefficients among environmental and water quality variables in a tilapia pond. Subfigure (a) presents Pearson correlation [27], suitable for linear relationships, revealing strong associations between air and water temperature (r = 0.95) and between pH and dissolved oxygen (r = 0.90), while humidity shows a strong negative correlation with temperature (r = −0.79). Subfigure (b) shows Spearman correlation, appropriate for nonlinear or non-normally distributed data, with similar trends observed—air temperature correlates with water temperature (ρ = 0.94), and pH remains highly associated with dissolved oxygen (ρ = 0.89) [28]. Using both Pearson and Spearman methods provides a comprehensive view of variable relationships across linear and monotonic patterns.

The Spearman correlation analysis revealed strong positive relationships among air temperature, water temperature, and light intensity (r = 0.94, 0.89, and 0.86, respectively), indicating the influence of solar radiation on thermal changes in both air and water. Relative humidity showed strong negative correlations with air temperature (r = −0.79) and water temperature (r = −0.81), reflecting increased evaporation under warmer conditions. Electrical conductivity (EC) was moderately correlated with temperature (r = 0.56) and water temperature (r = 0.59) but negatively correlated with humidity (r = −0.84), suggesting ion concentration increases as water evaporates. Additionally, water pH was highly correlated with dissolved oxygen (r = 0.89), highlighting the role of aquatic photosynthesis in oxygen generation [29]. Overall, the results underscore significant interrelationships between environmental conditions and water quality parameters in aquaculture systems.

### 4.3. Predictive Model Performance

#### 4.3.1. Dataset for Training and Testing the Model

The dataset used in this study consisted of 2208 hourly observations collected between June and August, capturing both environmental and water quality parameters for aquaculture systems. Five independent variables, namely air temperature (°C), relative humidity (%), light intensity (lux), water temperature (°C), and electrical conductivity (µS/cm), were selected as predictors, while two dependent variables, namely pH and dissolved oxygen (DO, mg/L), served as the prediction targets. This variable selection allows for a comprehensive representation of the complex interactions between external environmental factors and internal water quality conditions. The data were chronologically split into training (80%) and testing (20%) sets to prevent data leakage and ensure temporal integrity, which is a suitable approach for time-series modeling.

#### 4.3.2. Approach and Algorithms Employed

This study employed a comparative approach using three regression algorithms to forecast water quality parameters: Multiple Linear Regression (MLR), Random Forest Regression (RFR), and Decision Tree Regression (DTR). MLR provides interpretable linear relationships, RFR enhances predictive accuracy in complex, nonlinear data, and DTR offers transparent rule-based structures with a risk of overfitting. Model performance was evaluated using RMSE, MAE, and R^2^ metrics. All implementations were conducted in Python (version 3.12.11) via Google Colab, utilizing standard libraries including NumPy (2.0.2), Pandas (2.2.2), and scikit-learn (1.6.1) for efficient and reproducible analysis.

To maximize prediction performance, hyperparameter tuning was performed for tree-based models. For the Decision Tree Regressor (DTR), parameters including maximum depth (5, 10, unlimited), minimum samples split (2, 5, 10), and minimum samples per leaf (1, 2, 4) were tested. For the Random Forest Regressor (RFR), the number of estimators (50, 100, 200), maximum depth (5, 10, unlimited), and maximum features (“sqrt”, “log2”) were evaluated. Grid search with five-fold cross-validation was applied to select the optimal configuration based on RMSE minimization.

#### 4.3.3. Forecasting Results and Accuracy Evaluation

For DO prediction, model performance was compared across MLR, DTR, and RFR using chronologically split datasets. As shown in Table 4, MLR achieved R^2^ = 0.6438, RMSE = 0.3711, and MAE = 0.2999, indicating moderate accuracy with limited capacity for nonlinear patterns. DTR showed slightly lower R^2^ (0.6017) but improved MAE (0.2416), suggesting closer predictions in most cases, though higher RMSE (0.3924) pointed to some instability due to outliers. RFR outperformed both, with R^2^ = 0.8079, RMSE = 0.2725, and MAE = 0.1842, confirming its strength in capturing temporal and nonlinear relationships. Despite RFR’s superior accuracy, its computational cost makes it more suitable for server-side deployment. In contrast, MLR remains viable for lightweight on-device inference (e.g., ESP32).

While Table 4 presents baseline performance under a single train–test split, Table 5 reports the average performance across five-fold cross-validation with standard deviations. These results further confirm the robustness of the findings: RFR consistently achieved the highest accuracy and stability in predicting both DO and pH, with R^2^ values above 0.80 and the lowest error metrics. MLR maintained acceptable performance with lower computational demand, supporting its potential for edge-device deployment. DTR showed competitive results but exhibited greater variability, indicating sensitivity to data splits. Together, the two tables demonstrate not only the relative ranking of models but also the consistency of their performance under resampling strategies.

The final MLR model for DO prediction is shown in Equation (1). Notably, although light intensity was included among the input features, its regression coefficient was zero, indicating no significant linear contribution to the model under the training dataset.

(1) $$y_{DO}=3.8365-0.1197\left(ATemp\right)+0.0106(RH)+0.2319(WTemp)-0.001(EC)$$

For pH prediction, the RFR model demonstrated the highest performance with an R^2^ of 0.8327, RMSE of 0.1463, and MAE of 0.0974, indicating superior accuracy and stability. In comparison, the linear MLR model achieved an R^2^ of 0.7192, RMSE of 0.1896, and MAE of 0.1521. The DTR model showed a comparable R^2^ (0.7148) and a lower MAE (0.1160) than MLR, but with a slightly higher RMSE (0.1910). While RFR offers the best predictive performance, it requires higher computational resources, making it more suitable for server-based deployment. On the other hand, MLR remains a practical choice for field IoT applications, particularly when deploying models directly on microcontrollers. The MLR-based prediction model for pH can be expressed as Equation (2):

(2) $$y_{pH}=1.1629-0.241\left(ATemp\right)+0.021(RH)+0.3955(WTemp)-0.002(EC)$$

**Figure 11:**
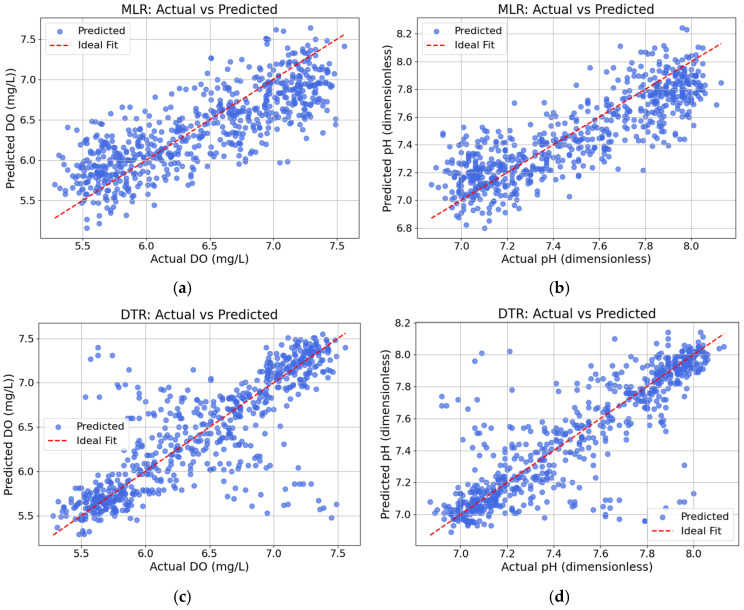

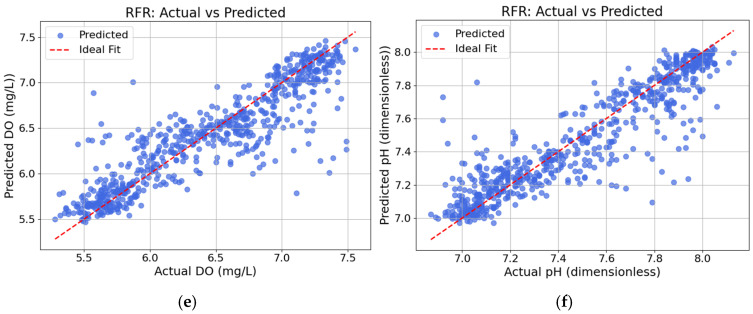
Comparison of actual and predicted values for DO (**a**,**c**,**e**) and pH (**b**,**d**,**f**) using MLR, DTR, and RFR models.

Figure 11 compares actual and predicted values for DO (a, c, e) and pH (b, d, f) across the MLR, DTR, and RFR models. MLR results (a–b) show that pH predictions align closely with the ideal line. DO predictions exhibit greater deviation, particularly at the extremes, reflecting MLR’s limitations in capturing nonlinear DO patterns. DTR predictions (c–d) improve accuracy, especially for pH, where data points cluster tightly along the ideal line. DO predictions, though better than MLR, still show some dispersion due to temporal or external variability. RFR (e–f) yields the most accurate results, with both DO and pH predictions densely following the ideal fit, indicating superior model stability, lower bias, and stronger handling of complex interactions.

### 4.4. Experimental Results of the Water Quality Prediction and Control System

**Figure 12:**
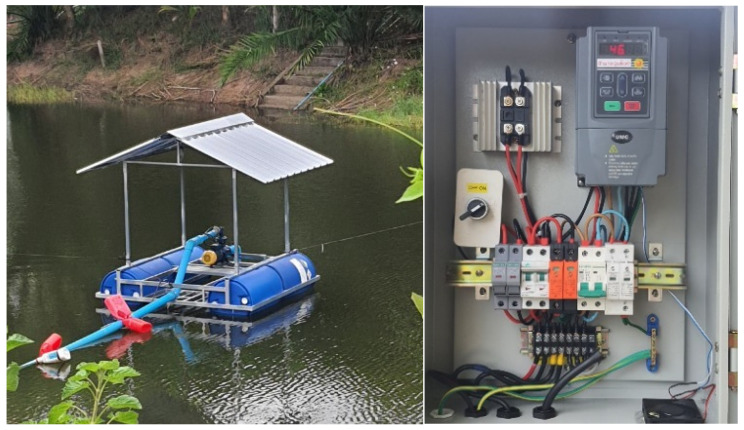
Water quality control system.

In this experiment, the Multiple Linear Regression (MLR) equations for predicting dissolved oxygen (DO) and pH were deployed on an ESP32 microcontroller. The code was designed to acquire environmental sensor data and compute DO and pH values directly from the regression model. This approach enables real-time water quality estimation without using physical DO and pH sensors, which are costly and require frequent calibration. As a result, system costs and maintenance are significantly reduced. The ESP32 can also connect seamlessly with IoT platforms such as Blynk or Google Sheets for data logging and visualization, as shown in Figure 12.

**Figure 13:**
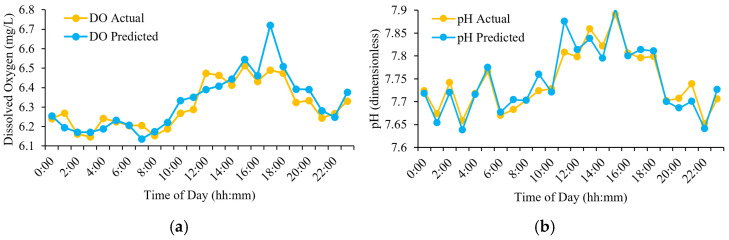
Hourly comparison between actual and predicted values of (**a**) DO and (**b**) pH.

As illustrated in Figure 13, the predicted values of DO and pH generated by the MLR-based forecasting model closely follow the actual sensor measurements throughout the 24 h cycle. This temporal alignment highlights the model’s robustness and reliability in capturing the diurnal fluctuations of water quality parameters. The consistency of prediction, particularly during peak environmental variation (e.g., 8:00–17:00), confirms the model’s suitability for real-time deployment on embedded microcontroller platforms, such as ESP32, in low-cost IoT water monitoring applications.

This experiment evaluated an automated water quality control system covering DO, pH, and localized temperature regulation. During 04:00–07:00, the system effectively prevented DO drops below 6 mg/L (100% prevention), with recovery from 6.0 to 6.4 mg/L averaging 38 min and energy consumption of only 0.32 kWh per cycle. The pH adjustment module reduced values from 8.25 to 7.95 within 45 min with a minimal overshoot of 0.04 and chemical usage of 0.35–0.55 L/100 m^3^. The spot-heating unit raised water temperature by 1.8 °C within 25 min, covering a 1.5–2.5 m radius using 0.85 kWh/h, while fish aggregation near the warm zone increased by up to 50%. Overall, the results demonstrate the system’s accuracy, efficiency, and potential for scalable IoT-based solutions in sustainable aquaculture ecosystem management. To statistically validate the observed effects, paired *t*-tests comparing conditions before and after interventions were performed. Results confirmed significant increases in DO after aeration (*p* < 0.001) and significant decreases in pH after acid dosing (*p* < 0.001), as summarized in Table 6.

Table 7 presents a side-by-side comparison between the MLR model deployed directly on an ESP32 microcontroller and the RFR model executed via a cloud/server setup. The MLR approach offers lightweight, real-time prediction with minimal resource requirements, suitable for low-cost IoT systems. In contrast, RFR achieves higher accuracy but requires greater computational resources and network infrastructure, making it more suitable for centralized or precision-critical applications.

## 5. Discussion

This study presents the design, development, and testing of an integrated water quality forecasting and control system for semi-intensive tilapia aquaculture in Northern Thailand. By combining IoT-based environmental sensing, edge computing, and statistical analysis, the research aimed to enhance real-time water quality management and provide insights into the interactions between environmental conditions and fish behavior. The key findings are organized into three main areas: environmental monitoring, data analysis, and the development of predictive models and automated control mechanisms.

### 5.1. Environmental Monitoring and Water Quality Assessment

Continuous monitoring of water quality parameters over three months (May–July 2025) revealed distinct seasonal patterns affecting pond ecology. A gradual decline in air temperature (from 31.5 °C to ~29 °C) aligned with increased rainfall, which concurrently lowered water temperature—impacting tilapia behavior, particularly their morning aggregation [30]. The rise in relative humidity (from 68–72% to 80–85%) during the rainy season corresponded with a drop in electrical conductivity (EC) from 300 to 200 µS/cm, indicating ion dilution from rainwater inflow [31]. These changes are ecologically significant as they alter the mineral balance essential for fish health.

The pH remained stable between 7.48 and 7.52, while dissolved oxygen (DO) levels increased from 6.30 to 6.55 mg/L, supporting favorable conditions for tilapia growth. These trends aligned with consistent light intensity (8000–11,000 lux) and increased photosynthetic activity toward the end of July [32]. The sensor network demonstrated reliable performance, providing a valuable dataset for subsequent machine learning applications aimed at predictive water quality control [33,34].

### 5.2. Data Analysis and Environmental Correlations

Effective data preprocessing, particularly the handling of missing and outlier values, proved essential for accurate analysis. The cleaned data reflected natural pond dynamics, with average values indicating optimal conditions for tilapia: water temperature ≈ 29 °C, EC ≈ 249.74 µS/cm, pH ≈ 7.5, and DO ≈ 6.43 mg/L. DO levels above 5 mg/L are critical for tilapia survival and growth [35].

Correlation analysis revealed strong positive relationships between air temperature and water temperature (r = 0.94) and light intensity (r = 0.89), consistent with solar energy transfer dynamics. Relative humidity negatively correlated with temperature (r ≈ −0.80), as expected from meteorological principles. A notable correlation between DO and pH (r = 0.89) likely reflects phytoplankton photosynthesis, which increases both oxygen levels and pH during daylight hours [36].

In developing machine learning models for water quality forecasting, MLR offered transparency and low computational cost, ideal for IoT devices like the ESP32. However, its linear nature limits its ability to model complex relationships. While DTR provided better nonlinearity handling, it was prone to overfitting without proper tuning. RFR emerged as the most accurate and stable model, with R^2^ > 0.80 and the lowest RMSE and MAE values for both DO and pH [37,38]. These results underscore the need to balance model accuracy with hardware constraints—favoring RFR for cloud/server deployment, and MLR for resource-constrained, edge-level applications.

Recent related studies using hybrid or deep learning models have achieved even higher accuracy. For example, Hu et al. [39] developed an RBF neural network optimized via Grey Relational Analysis (GRA) and demonstrated R^2^ ≈ 0.96 for aquatic production forecasting, outperforming BP, GA-BP, and LSTM models in the same study. These methods highlight the potential of advanced models for capturing nonlinear and regional temporal patterns, but their computational and connectivity demands make them less practical for smallholder aquaculture. In contrast, the present ESP32-based system prioritizes feasibility and offline functionality, providing a trade-off between predictive power and real-world applicability.

### 5.3. Predictive Control and System Performance

Experimental validation of the integrated prediction control system using MLR on ESP32 showed that even resource-limited devices can effectively compute linear models for real-time DO and pH prediction, eliminating the need for expensive sensors and reducing installation and maintenance costs [40]. This approach aligns with Sharafi et al., who proposed low-cost, resilient IoT forecasting systems using lightweight models for remote aquaculture [40]. The present results validate this concept for practical farm-level deployment.

Automated control mechanisms responded accurately to predicted values. The aeration system maintained DO levels between 6.2 and 6.4 mg/L using only 0.32 kWh per cycle, demonstrating high energy efficiency. These findings are consistent with recent work by Mao et al. [41], who highlighted the importance of accurate DO monitoring for sustainable aquaculture management, using satellite-based machine learning to retrieve DO concentrations in fishponds. The pH control system successfully stabilized levels within a safe range (7.9–7.98) using minimal acid addition, preventing overshoots. Meanwhile, the localized heating system stimulated fish aggregation behavior by 35–50%, facilitating efficient feeding and health monitoring. In this prototype, the heating experiment was intended primarily as a proof-of-concept to demonstrate the feasibility of localized thermal control rather than to provide a comprehensive energy cost–benefit analysis. While the behavioral benefits were evident, we recognize that under limited solar or battery supply, trade-offs between additional energy consumption and aggregation outcomes must be carefully considered. This issue has therefore been explicitly noted as a direction for future work.

The threshold-based on/off logic used in this prototype shares conceptual similarities with finite state machine (FSM) control strategies, which explicitly incorporate thresholds and hysteresis in a structured manner [42]. Although our implementation was intentionally simplified to reduce computational burden on the ESP32, recognizing this connection strengthens the theoretical foundation of the control approach. Future work may further formalize this logic by integrating FSM-based design principles or by adopting explicit hysteresis bands or PID-style controllers to enhance stability and prolong actuator lifespan.

These outcomes not only confirm the technical feasibility but also highlight potential economic benefits for farmers. Energy-efficient aeration and targeted pH dosing reduce operational costs through lower electricity and chemical use, while improved fish aggregation and survival rates enhance productivity and income stability. The adoption of low-cost ESP32 devices further supports accessibility for smallholder farmers, linking technological innovation with long-term economic sustainability.

In the long term, the proposed framework envisions reducing dependency on continuous DO and pH sensing by using predictive models as the primary source of water quality estimation. Physical DO/pH probes would be retained only as backup instruments for periodic validation, thereby extending sensor lifespan and lowering operational expenses.

In summary, this study demonstrates the potential of combining IoT sensing, machine learning, and automation to achieve low-cost, sustainable water quality management in aquaculture. The MLR-on-Edge approach proves viable for small-scale farms, while higher-accuracy models like RFR are suited for centralized cloud processing. The proposed system supports environmental, economic, and energy efficiency goals, aligning with the FAO’s vision of “Smart Aquaculture,” which advocates for AIoT adoption to enhance long-term sustainability in aquaculture systems [43]. Several limitations, however, should be acknowledged. The present dataset only spans May–July 2025 (rainy season), restricting the generalizability of predictions to dry or cold seasons. Differences in pond types, hydrochemical profiles, and regional climatic conditions were also not addressed, which may affect transferability. In addition, aquaculture environments are inherently dynamic, raising the issue of model drift. To overcome these challenges, future work will extend data collection across multiple seasons and sites, while exploring transfer learning and federated learning approaches to improve scalability and adaptability. Model retraining will be pursued through a hybrid edge–cloud strategy, where updated models are periodically refined offline and redeployed to the ESP32. Moreover, more advanced control strategies, such as explicit hysteresis bands, FSM-based designs, or PID-style controllers, will be investigated to enhance long-term stability and actuator lifespan. Furthermore, while regression- and ensemble-based machine learning models were the main focus of this study, nonlinear Kalman filtering and other state-estimation techniques might also produce reliable and computationally effective forecasts on embedded devices. Future research will incorporate comparative evaluations to assess their trade-offs in accuracy, complexity, and resource requirements.

## 6. Conclusions

This research presented the development and deployment of an Edge AI system for water quality monitoring and control in small-scale tilapia aquaculture, utilizing the low-cost ESP32 microcontroller. The system was designed to perform real-time environmental sensing, parameter prediction (DO and pH), and control, all without the need for constant cloud connectivity. It successfully collected and analyzed key environmental variables such as air temperature, humidity, EC, and light intensity with clear seasonal patterns, including decreasing temperatures and rising humidity during the rainy season, which affected water temperature and ion concentration. Correlation analysis revealed meaningful biological relationships, such as the positive association between pH and DO driven by phytoplankton photosynthesis during daylight hours.

Although the RFR model showed higher predictive accuracy, the MLR model demonstrated practical advantages for embedded deployment on ESP32, with low resource consumption and offline operability. Field experiments confirmed that the system could reliably maintain DO levels within safe ranges, adjust pH effectively, and even influence fish behavior through localized heating. The system also exhibited energy efficiency and environmental friendliness, aligning with sustainable aquaculture goals.

In summary, the proposed Edge AI system fulfills technical requirements for real-time water quality monitoring and control. Also, it shows strong practical significance for rural aquaculture by reducing operational costs, minimizing risks, and enabling adoption in areas with unstable connectivity. Despite current constraints such as limited seasonal coverage and the linear nature of the deployed model, future expansions involving multi-season data, federated learning approaches, and economic performance analysis could enhance the system’s robustness and scalability for broader commercial and community use. Specifically, the present study covered only three months (May–July), corresponding to part of the rainy season in Northern Thailand. Therefore, seasonal variations in dry and cold periods were not captured, which may reduce generalizability. Future work will extend data collection across multiple seasons to ensure that the system remains robust under diverse environmental conditions. In addition, future studies will consider more advanced control strategies, such as introducing explicit hysteresis bands or PID-style control, to further improve system stability and extend actuator lifespan.

## Figures and Tables

**Table 1:** Summary of IoT and ML-based water quality studies.

Study	Use of IoT	ML Models Used	Computation Level	Implementation
IoT Real-Time Potable Water Quality Monitoring and Prediction Model [11]	Yes	Decision tree, SVM, Random Forest, gradient boosting, neural network	Cloud-based (Arduino data sent via NB-IoT)	Monitoring; prediction of drinkability, alerts via text; no control
Intelligent Edge-Cloud Framework for Water Quality Monitoring in Water Distribution System [12]	Yes	Deep-learning classification (unspecified models)	Edge, cloud, and hybrid scenarios	Classification: compares latency, throughput, and accuracy; monitoring only
Dissolved Oxygen Forecast Model Using Hybrid Machine Learning [13]	No	Hybrid MODWT-MARS; compares Random Forest, SVR, KNN, KRR	Server-based	Predicts DO using hydro-meteorological data; no IoT
Short-Term Prediction of Dissolved Oxygen in a Microtidal Estuary [14]	No	RNN, LSTM, MLP, gradient boosting, AutoKeras	Server-based	Predicts DO using historical data and weather; no IoT
Integrated Smart Pond Water Quality Monitoring and Fish Farming Recommendation AquaBot System [15]	Yes	Ensemble model (bagging/boosting/stacking), Random Forest, SVM, decision tree, KNN, logistic regression	Cloud-based (ESP-32 data stored on Google Sheets/Firebase)	Recommend fish species; monitoring; no water quality control
Random Forest-Based Water Quality Prediction Framework for Inland and Nearshore Water Bodies [16]	No	Random Forest (compared with SVR, KNN, MLP, GBRT, bagging)	Server/cloud (analysis using sensors and remote sensing)	Predicts TN and salinity; monitors but does not control

**Table 2:** Missing data summary before and after preprocessing.

Variable	Missing Count (Before)	Missing % (Before)	Missing Count (After)	Missing % (After)
Temperature	95	4.28	0	0.0
Humidity	66	2.97	0	0.0
LightIntensity	79	3.56	0	0.0
waterTemp	100	4.51	0	0.0
EC water	67	3.02	0	0.0
pH water	65	2.93	0	0.0
Oxygen water	65	2.93	0	0.0

**Table 3:** Descriptive statistics of environmental and water quality variables.

Index	Atemp (°C)	RH (%)	Light (lux)	Wtemp (°C)	EC (µS/cm)	pH	DO (mg/L)
count	2208.0	2208.0	2208.0	2208.0	2208.0	2208.0	2208.0
mean	30.03	78.25	8990.86	29.00	249.74	7.50	6.43
std	5.13	9.85	11,806.96	3.98	47.44	0.35	0.63
min	19.8	52.43	0.0	21.8	147.47	6.87	5.28
25%	25.24	71.69	0.0	25.2	215.27	7.15	5.83
50%	29.94	77.93	725.88	28.99	249.97	7.50	6.43
75%	34.77	85.88	17,399.07	32.80	284.90	7.85	7.03
max	40.21	100.0	46,161.43	36.12	349.33	8.14	7.59

**Table 4:** Comparison of MLR, DTR, and RFR for DO and pH.

Model	DO			pH		
	RMSE	MAE	R^2^	RMSE	MAE	R^2^
MLR	0.3711	0.2999	0.6438	0.1896	0.1521	0.7192
DTR	0.3924	0.2416	0.6017	0.1910	0.1160	0.7148
RFR	0.2725	0.1842	0.8079	0.1463	0.0974	0.8327

**Table 5:** Cross-validation (5-fold) results for MLR, DTR, and RFR models predicting DO and pH.

Model	DO			pH		
	RMSE (±SD)	MAE (±SD)	R^2^ (±SD)	RMSE (±SD)	MAE (±SD)	R^2^ (±SD)
MLR	0.386 ± 0.03	0.311 ± 0.02	0.624 ± 0.05	0.191 ± 0.02	0.154 ± 0.01	0.711 ± 0.03
DTR	0.387 ± 0.05	0.232 ± 0.04	0.623 ± 0.07	0.198 ± 0.03	0.118 ± 0.02	0.688 ± 0.04
RFR	0.273 ± 0.03	0.185 ± 0.02	0.812 ± 0.04	0.146 ± 0.02	0.097 ± 0.01	0.832 ± 0.03

**Table 6:** Paired *t*-test results for DO and pH.

Variable	Condition	n	Mean ± SD	Paired Diff (Mean ± SD)	t (df)	*p*
DO (mg/L)	Before vs. after aeration	12	6.03 ± 0.18 → 6.38 ± 0.17	+0.35 ± 0.14	8.64 (11)	<0.001
pH	Before vs. after acid dosing	12	8.26 ± 0.09 → 7.96 ± 0.08	−0.30 ± 0.10	9.49 (9)	<0.001

**Table 7:** Edge vs. cloud models for water quality forecasting.

Criterion	MLR on ESP32	RFR on Cloud/Server
Model Type	Linear Regression (MLR)	Ensemble Decision Tree (RFR)
Prediction Accuracy (pH)	R^2^ = 0.7192, RMSE = 0.1896, MAE = 0.1521	R^2^ = 0.8327, RMSE = 0.1463, MAE = 0.0974
Prediction Accuracy (DO)	R^2^ ≈ 0.6800 (example), lower nonlinear handling	R^2^ ≈ 0.7900–0.8100, better at capturing dynamics
Computational Resource	Low (runs on microcontroller)	High (requires CPU/GPU or server/cloud)
Hardware Requirement	ESP32 only	ESP32 + server/cloud connection
Real-Time Capability	Yes (fully local and fast)	Yes, but depends on network latency
Power Consumption	Minimal (within ESP32 limits)	Higher (includes server/network energy)
Scalability and Maintenance	Easy to deploy in remote areas	Requires IT infrastructure and maintenance
Cost Efficiency	Very low (no DO/pH sensors, no cloud)	Higher (cloud service, sensors optional but useful)
Best Use Case	Edge computing, cost-sensitive IoT deployments	Precision-critical systems with backend support

## Data Availability

The raw data supporting the conclusions of this article will be made available by the authors on request.
